# Healthy Habit Adherence in Uruguayan Women Under Breast Cancer Follow-Up

**DOI:** 10.7759/cureus.82621

**Published:** 2025-04-20

**Authors:** Natalia Camejo, Cecilia Castillo, Dahiana Amarillo, Guillermo Caraballo, Florencia Fernandez, Maria Pia Fanco, Mauro Gazzola, Valeska Piñeiro, Serena Rodriguez, Guadalupe Herrera, Gabriel Krygier

**Affiliations:** 1 Department of Oncology, Hospital de Clinicas, Montevideo, URY; 2 Department of Clinical Oncology, Hospital de Clinicas, Montevideo, URY; 3 Department of Clinical Oncology, Hospital de Clínicas "Dr. Manuel Quintela", Montevideo, URY; 4 Department of Clinical Oncology, School of Medicine, University of Uruguay, Montevideo, URY; 5 Department of Preventive and Social Medicine, School of Medicine, University of Uruguay, Montevideo, URY

**Keywords:** breast cancer, healthy habits, lifestyle intervention, quality of life (qol), uruguay

## Abstract

Introduction: Breast cancer (BC) ranks first in incidence and mortality among Uruguayan women. Healthy habits (HHs) - maintaining a healthy weight, engaging in regular physical activity, consuming fruits and vegetables (F&V), avoiding smoking, and limiting alcohol intake - are associated with better quality of life and reduced risk of recurrence. However, there is limited information on the prevalence of HH among Uruguayan women with BC.

Methods: This observational, descriptive, multicenter study included patients with stage I-III BC from public and private healthcare centers in Uruguay. Adherence to HH was assessed using a questionnaire based on the American Cancer Society guidelines, evaluating diet, physical activity, alcohol, and tobacco use. Patients were classified into low (0-2), moderate (3), and high adherence (4-5) groups. Associations with specific variables such as healthcare setting (private vs. public), age, cancer stage, and time since diagnosis were analyzed using the Chi-squared test.

Results: A total of 209 patients were included. Overall, 80.8% (169 patients) adhered to three or more HH. Higher adherence was observed among patients attending private centers (89.6%, 69 patients) compared to public ones (75.7%, 100 patients; p=0.014). The most commonly met habits were non-smoking (88.9%; 95% CI: 84.0%-92.6%), low alcohol intake (99.0%; 95% CI: 96.6%-99.7%), and body mass index (BMI) <30 kg/m² (68.9%; 95% CI: 62.3%-74.8%). Lower adherence was seen for sufficient physical activity (55.5%; 95% CI: 48.7%-62.1%) and adequate F&V intake (31.6%; 95% CI: 25.7%-38.2%). No significant differences were found in adherence according to age, stage, or time since diagnosis.

Conclusion: Although 80.8% of patients adhered to at least three HHs, critical areas for improvement remain, particularly physical activity and F&V consumption, especially in the public sector. These findings highlight the need for targeted strategies to promote HH in BC patients to improve long-term outcomes and quality of life. Results should be interpreted with caution due to the use of self-reported questionnaires and the non-probabilistic nature of the sample.

## Introduction

Breast cancer (BC) is the most prevalent type of cancer among women worldwide and represents a significant challenge for both public health and personalized medical care [1]. Although advances in early detection and treatment have increased survival rates [2,3], survivors face a high risk of recurrence, comorbidities, and associated mortality, largely attributable to modifiable lifestyle factors [4-7]. Several studies have shown that adopting healthy habits (HHs) - such as maintaining a balanced diet, engaging in regular physical activity, and avoiding risk behaviors (e.g., smoking and excessive alcohol consumption) - can significantly improve outcomes in terms of quality of life, overall survival, and reduced risk of recurrence [8-14]. These data, combined with the growing number of cancer survivors worldwide - over 22 million according to recent estimates [1,2] - have sparked renewed interest in investigating health-related behaviors in this population. International studies show that despite the known benefits of HH, many cancer survivors continue to engage in risk behaviors similar to those of the general population, representing a key opportunity for implementing health promotion strategies [4-8]. Nevertheless, adherence rates to these recommendations, based on the American Cancer Society (ACS) guidelines [15], remain low among BC survivors [16-20]. This low adherence may be influenced by structural and sociocultural barriers, such as limited access to health resources, economic inequality, and lack of information or support to adopt these habits. For example, it has been reported that only 37.1% of patients meet the recommendation of at least 150 minutes of moderate or vigorous physical activity per week, and just 18.2% consume the recommended five servings of fruits and vegetables (F&V) per day [21]. Furthermore, only 5% of cancer survivors meet all three main recommendations (physical activity, healthy diet, and not smoking), highlighting the need for comprehensive interventions to improve these rates and, in turn, the quality of life of patients [21].

In Uruguay, information regarding HHs among cancer survivors is scarce. A recent study assessed adherence to HH among survivors receiving care in two public institutions and found that although most patients refrained from smoking (80.6%) and moderated alcohol intake (95.5%), less than 15% met the recommended intake of F&V, and only 32.3% engaged in sufficient physical activity [22]. These findings highlight significant disparities in HH adherence and underscore the need for more targeted research that includes different types of cancer and considers factors such as the type of healthcare institution. The present study focuses exclusively on BC survivors and expands on previous work by incorporating data from both public and private institutions. Its main objective is to characterize HH adherence in this population and explore its association with clinical and sociodemographic variables. This analysis will contribute to scientific knowledge and help identify key areas to enhance equity in health promotion, particularly among BC survivors receiving care in diverse socioeconomic settings.

In this context, we aimed to analyze the extent to which Uruguayan patients under follow-up for BC adhere to the HHs recommended by the ACS, by evaluating the proportion of adherence and its association with clinical and sociodemographic variables. We were particularly interested in exploring whether differences exist between patients treated in the public versus the private sector. We hypothesized that overall adherence to HHs is suboptimal, especially regarding physical activity and F&V intake, and that adherence levels differ by type of healthcare institution, with higher adherence expected among patients treated in the private sector, possibly due to better availability of resources and opportunities to adopt these habits.

## Materials and methods

Objectives

Primary objective is to assess the prevalence of HHs among patients under follow-up for BC, defined as: consuming F&V at least five times per day, maintaining a body mass index (BMI) below 30 kg/m², engaging in at least 150 minutes of physical activity per week, not smoking, and limiting alcohol consumption.

The secondary objective is to evaluate whether HH adherence varies according to age group (≤65 vs. >65 years), type of healthcare center (public vs. private), time since diagnosis (≥5 years vs. <5 years), and cancer stage.

Methods

This was a cross-sectional, descriptive, multicenter observational study conducted between March and June 2024. Patients included were women over 18 years old, diagnosed with BC stage I-III, who had completed chemotherapy and/or radiotherapy more than one year prior and were under routine follow-up care. Patients receiving hormone therapy at the time of the study were eligible. Those undergoing active chemotherapy or radiotherapy were excluded due to the potential temporary impact of these treatments on lifestyle habits (e.g., diet, physical activity, work). In contrast, hormone therapy - given its long duration and low toxicity - generally allows patients to resume daily activities and maintain healthy behaviors.

Patients were identified through the medical records of the oncology departments of the participating institutions: Hospital de Clínicas, Servicio Médico Integral (SMI), Hospital Departamental de Soriano, and Hospital Británico. Patients with scheduled appointments during the inclusion period were invited to participate during their visit. Those who agreed provided written informed consent and then completed the questionnaire. Patients without scheduled appointments during the inclusion period were contacted by phone; if they expressed interest in participating, verbal consent was obtained, and the survey was completed via phone.

HHs were evaluated based on the 2022 updated guidelines from the ACS [15]. F&V consumption was considered adequate if patients consumed at least five servings per day, excluding fried potatoes. BMI was classified as normal (<25 kg/m²), overweight (25-29.9 kg/m²), or obese (≥30 kg/m²); adherence was defined as a BMI <30 kg/m². Physical activity was considered adequate if patients reported engaging in at least 150 minutes of moderate or 75 minutes of vigorous activity per week. Regarding tobacco use, patients who had smoked more than 100 cigarettes in their lifetime and were still smoking were classified as non-adherent. Alcohol intake was assessed in terms of frequency and quantity, and adherence was defined as consumption of fewer than one drink per day for women and fewer than two for men in the last 30 days.

An ad hoc questionnaire was developed for data collection based on the ACS recommendations. The content was reviewed by experts in oncology and public health, and a pilot test was conducted with a small sample of patients (n=10) to evaluate clarity, comprehension, and cultural appropriateness. The questionnaire was completed either in person or by phone. Additionally, clinical records were reviewed to collect data on age, cancer stage, diagnosis date, and treatment received. Educational materials about the importance of following these recommendations were provided to participants.

Each patient received a healthy behavior score by assigning 0 for non-adherence and 1 for adherence to each of the five behaviors: F&V intake, BMI, physical activity, smoking, and alcohol use. These scores were summed to create a total score ranging from 0 to 5. Based on this score, patients were grouped into low adherence (scores 0-2), moderate adherence (score of 3), and high adherence (scores 4-5). This classification was based on prior studies using cumulative scoring systems of five healthy behaviors in oncology populations, with one point assigned per behavior met. Grouping into three levels (low, moderate, and high) allows identification of subgroups with varying degrees of adherence and is a common approach in recent literature on cancer survivors [17].

Descriptive statistics were used for continuous variables to characterize the study population. HH adherence was estimated by calculating proportions and corresponding confidence intervals. To assess associations between adherence and specific variables, the Chi-squared test was used for categorical variables, including healthcare setting (public vs. private), current age (≤65 vs. >65 years), time since diagnosis (<5 vs. ≥5 years), and cancer stage (I, II, or III). Statistical significance was defined as p<0.05, with the null hypothesis being independence between variables. Analyses were performed using Jeffreys’s Amazing Statistics Program (JASP) software version 0.19.1.0 (University of Amsterdam, Netherlands), allowing calculation of observed and expected frequencies, as well as proportions and their confidence intervals. This approach enabled the identification of categories with significantly different levels of adherence.

The study protocol was approved by the Research Ethics Committee of the Hospital de Clínicas (Ref. 48-24E), in compliance with the Declaration of Helsinki and national regulations (Law No. 18.335 and associated decrees). Confidentiality measures included assigning anonymous codes to each patient and securely storing collected data on restricted-access devices. Only the research team had access to the database, which was used exclusively for scientific purposes.

## Results

Out of a total of 230 patients who met the inclusion criteria, 209 agreed to participate and completed the questionnaire. The median age was 65 years, ranging from 22 to 87 years. Of the total sample, 63% (132 patients) were treated in the public sector, and 37% (77 patients) in the private sector. Regarding cancer stage at diagnosis, 38.7% (81 patients) were diagnosed with stage I, 43.1% (90 patients) with stage II, and 18.2% (38 patients) with stage III. The remaining clinicopathological characteristics are shown in Table 1.

**Table 1:** Clinicopathological characteristics of the patients (n=209)

Variable	Category	n	Percentage
Current age	≤ 65 years	105	50
	> 65 years	104	49.76
Cancer stage	I	81	38.76
	II	90	43.06
	III	38	18.18
Years since diagnosis	≥ 5 years	96	45.93
	< 5 years	113	54.07
Healthcare setting	Public	132	63.15
	Private	77	36.85

Regarding compliance with HH recommendations, 31.6% of patients (66 participants) met the guideline of consuming at least five daily servings of F&V (95% CI: 25.7%-38.2%). Concerning BMI, 68.9% of patients (n=144) had a BMI below 30 kg/m² (95% CI: 62.3%-74.8%). Among them, 35.4% (74 patients) were classified as having normal weight, 33.5% (70 patients) were overweight, and 31.1% (65 patients) were obese at various grades.

In terms of physical activity, 55.5% of patients (116 participants) met the recommendation of engaging in at least 150 minutes of moderate or 75 minutes of vigorous physical activity per week (95% CI: 48.7%-62.1%). With regard to tobacco use, a high proportion (88.9% (n=186)) reported not smoking (95% CI: 84.0%-92.6%). As for alcohol consumption, only 0.9% (n=2) reported excessive intake. Additionally, 67.5% (141 patients) reported abstaining completely, and 31.6% (66 patients) consumed less than one drink per day. When considering solely the recommendation to avoid excessive alcohol use, 99.0% of participants complied with this goal (95% CI: 96.6%-99.7%) (Table 2).

**Table 2:** Proportion of patients meeting each healthy habit, with 95% confidence intervals

Healthy habit	n (%)	95% CI
Sufficient physical activity	116 (55.5)	48.7 – 62.1
Adequate fruit and vegetable intake	66 (31.6)	25.7 – 38.2
Non-smoker	186 (88.9)	84.0 – 92.6
No excessive alcohol consumption	207 (99.0)	96.6 – 99.7
BMI < 30 kg/m²	144 (68.9)	62.3 – 74.8

A total of 19.2% of patients (40 participants) showed low adherence, meeting between 0 and two of the five recommended guidelines. In contrast, 23.6% (49 participants) demonstrated moderate adherence, fulfilling three recommendations, while 57.2% (120 participants) achieved high adherence, complying with four or five of the recommended behaviors.

When analyzing adherence by type of healthcare institution, patients followed in private centers showed higher adherence (89.6%, 69 participants) compared to those in public centers (75.7%, 100 participants). These patients met three or more HH recommendations, and the difference was statistically significant (p=0.014). This difference was particularly evident for F&V consumption (p=0.007), BMI (p=0.002), and physical activity (p=0.036). No statistically significant differences were observed regarding age groups (≤65 vs. >65 years) or time since diagnosis (<5 vs. ≥5 years), with p-values >0.05 in both cases.

## Discussion

This is the first study in Uruguay to assess the prevalence of HH among BC patients under follow-up care. A high prevalence of HH adherence was observed, with 80.8% (69 participants) meeting three or more recommendations. This may be attributable to ongoing prevention and awareness campaigns targeting both the general population and BC patients specifically [23-26]. Our findings show both similarities and differences when compared to previous data on cancer survivors treated in public institutions in Uruguay. In that study, only 28.5% of participants achieved high HH adherence, with particularly low compliance in F&V consumption (15%) and physical activity (33.2%) [22]. In contrast, our analysis, which included private institutions and focused exclusively on women, found a higher proportion of high adherence (57.2%, 120 patients), with 31.6% (66 patients) meeting the recommended F&V intake and 55.5% (116 patients) adhering to physical activity guidelines. These differences could reflect specific characteristics of the population included in our study, as well as greater resource availability in private healthcare settings. Furthermore, the previous study reported significantly higher adherence among women compared to men across nearly all evaluated categories. This may partly explain the higher adherence observed in the present study, which included only female patients. These results highlight the importance of considering contextual variables, such as healthcare setting and sociodemographic factors, when evaluating adherence to HH. They also underscore the need to develop tailored interventions for each setting, with particular attention to vulnerable populations served in public healthcare centers.

Our findings regarding F&V consumption (31.6%, 66 patients), physical activity (55.5%, 116 patients), and tobacco use (11.1%, 23 patients) are similar to those reported in an international study conducted in the United States. That study analyzed 7,443 female BC survivors with a median age of 64.2 years and an average survivorship duration of 7.4 years. Based on nationally representative data from the Behavioral Risk Factor Surveillance System (BRFSS) 2009 survey, adherence rates were 33.9% for adequate F&V intake, 53.8% for physical activity, and 10.2% for current smoking [16]. This comparison helps contextualize our findings within a similarly aged population, while acknowledging design and sociocultural differences between Uruguay and the United States. It is also worth noting that physical activity adherence in our study was higher than the 35% reported in a study of Hispanic and Latina BC survivors in the United States [27] and the 37% observed among healthy Hispanic and Latina women in the same country [28].

On the other hand, the prevalence of overweight and obesity in our study (64%, 135 patients) was significantly higher than the 24.7% reported in an international study conducted in the United States using data from the national BRFSS 2009 survey on female BC survivors [16]. This difference may be explained by structural and sociocultural factors specific to our country, such as lower availability and accessibility of healthy foods, differences in dietary patterns, less time allocated to physical activity, and economic inequalities that limit access to recreational spaces or preventive programs. Given that obesity is associated with poorer prognosis, increased risk of recurrence, and higher mortality [29], it is essential to implement educational and awareness strategies targeting this population to improve their quality of life. Furthermore, public policies could be developed, such as national campaigns promoting healthy eating and physical activity tailored to women with a history of BC.

At the institutional level, it would be beneficial to include nutritional assessments and physical activity counseling in follow-up oncology consultations, as well as to encourage collaboration with primary care teams. At the community level, partnerships with local markets could help improve access to fresh F&V, and programs offering free or low-cost physical activity opportunities could be promoted, particularly in the public sector. These multidisciplinary measures could help overcome both individual and structural barriers, contributing to greater equity in healthcare access and health outcomes.

However, it is important not to analyze BMI in isolation, but rather in conjunction with other behaviors and individual factors for each patient. In contrast, the prevalence of excessive alcohol consumption was markedly lower in our sample (<1%, two patients) compared to the 6.8% reported at the international level [17]. 

Finally, greater adherence to HH was observed among patients treated in the private sector, particularly regarding BMI, F&V consumption, and physical activity. This aligns with international data linking higher socioeconomic status to greater adherence to HH recommendations, likely due to better access to resources such as healthy foods and exercise programs [17]. These findings are also consistent with national data showing that both educational and economic level are directly associated with adherence to physical activity and other healthy behaviors [22].

This study presents several strengths that support the validity and relevance of its findings. First, it includes patients from various healthcare centers - both public and private - in Montevideo and other regions of the country, capturing a population with socioeconomic and cultural diversity and enhancing the generalizability of the results. Additionally, the sample size is substantial, which contributes to the robustness and statistical power of the conclusions. By aligning with the ACS guidelines, the study ensures that its assessment is consistent with international standards for the promotion of healthy behaviors, reinforcing its global relevance and its applicability to public health strategies.

However, this study presents several limitations that should be considered when interpreting the findings. First, it only describes the current HH of BC patients in follow-up and does not evaluate their impact on clinical outcomes. Therefore, it does not attempt to establish causal relationships between reported habits and prognosis or disease evolution. Additionally, the data were collected through self-administered surveys, which may introduce social desirability bias and recall errors, potentially affecting data accuracy. Another limitation is the lack of information on patients’ pre-diagnosis behaviors and how they may have changed throughout the disease trajectory.

While the study included participants from both public and private institutions, the sample was non-probabilistic and limited to four centers, which may reduce its generalizability at the national level. Voluntary participation could have introduced a self-selection bias, as patients who were more motivated or health-conscious may have been more likely to respond. Therefore, the results should be interpreted with caution, especially in terms of their generalizability to the broader population of BC survivors in Uruguay or to other populations with different contextual characteristics.

Based on the findings discussed, further research should be conducted to explore in greater depth the factors that hinder the adoption of certain HH, such as regular physical activity and adequate intake of F&V, which showed particularly low adherence rates in this population. It would be important to investigate individual and contextual perceptions, barriers, and facilitators-especially in public healthcare settings, where adherence was lower. It would also be valuable to develop and evaluate educational or community-based interventions aimed at improving adherence, and to assess their impact on quality of life and disease recurrence. These results may also serve as a foundation for designing more targeted public health prevention and promotion strategies that address the structural inequalities identified.

## Conclusions

This study shows that although a significant proportion of BC patients adhere to three or more recommended HH, critical areas remain that require attention, particularly the low intake of F&V and the high prevalence of overweight and obesity, especially in the public healthcare sector. Strengthening health promotion strategies and designing targeted public health policies has the potential to reduce disparities and improve health outcomes. In particular, these findings may serve as a foundation for developing national programs focused on education and support for healthy lifestyles, integrated into oncology follow-up care, with an emphasis on improving access to resources for physical activity and healthy eating among vulnerable populations. The cross-sectional design limited our ability to assess changes in HH before and after BC diagnosis. Future longitudinal studies are needed to evaluate these transitions and their associated factors. Additionally, conducting comparative studies between women with and without BC could help identify differences in health behaviors and specific needs of each group, providing essential evidence for the development of targeted interventions that could help reduce the burden of disease - particularly in a country like Uruguay, where BC remains the leading cause of cancer-related death among women.
